# Life-threatening biopsy of an iliopsoas pseudotumour in a patient with haemophilia: a case report

**DOI:** 10.1186/1752-1947-2-135

**Published:** 2008-04-30

**Authors:** Azan S Al Saadi, Ali H Al Wadan, Sami A El Hamarneh, Mohamed E Emad

**Affiliations:** 1 Department of Surgery, Sana'a University, Sana'a, Yemen; 2 Radiology Department, Alexandria University, Alexandria, Egypt

## Abstract

Iliopsoas pseudotumour is a serious complication of haemophilia. We present the case of a 20-year-old male patient with a six-month history of left leg weakness, limitation of movement and wasting of the muscles. Clinically he was diagnosed as having a psoas muscle rhabdomyosarcoma. During a computed tomography (CT) scan-guided Tru-cut biopsy he developed a serious and life-threatening bleeding from a retroperitoneal muscular haematoma. The patient underwent laparotomy prior to his final diagnosis of an Iliopsoas pseudotumour, which is a serious, as well as rare, complication of haemophilia.

## Introduction

Haemophilia A, an X-linked recessive bleeding disorder, is the most common severe type of inherited bleeding disorder, affecting 1 in 10,000 people. Although transmitted as a sex-linked disorder largely affecting males, it has been shown that 25% of all cases of haemophilia A arise by spontaneous mutation. The disorder is attributable to decreased blood levels of properly functioning procoagulant Factor VIII. The severity of the disease depends on the level of circulating clotting Factor VIII and is characterized by prolonged clotting time and partial thromboplastin time; the platelet count, platelet function tests and bleeding time are all within the normal range.

The clinical presentation of the disease depends on the circulating levels of FactorVIII and is categorized as mild, moderate or severe. Patients with haemophilia A often give a history of skin bruising, joint swelling and unusual bleeding associated with minor trauma or surgical procedures. The disease, however, may remain undetected without such history. This paper describes such a case where there were no previous episodes of joint swelling or bleeding. The haemophilia remained undetected until a biopsy was performed from psoas muscle tumour. The aim of this paper is to stress the importance of proper history and investigation prior to any invasive procedure, even if it is minor.

## Case presentation

**Figure 1:**
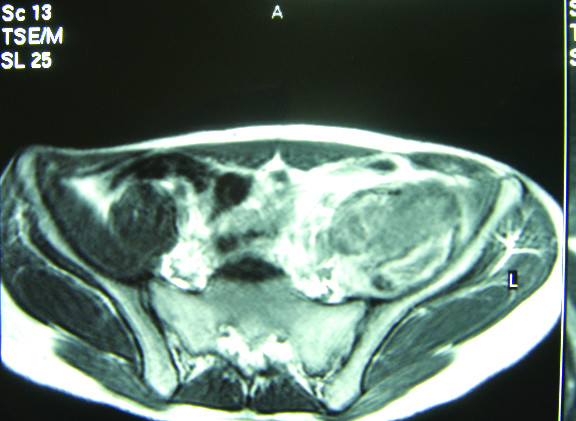
**MRI scan**. T1; Axial image, diffuse swelling is seen involving the left iliopsoas muscle showing heterogeneous signal intensity being iso-, hypo- and hyper-intense mostly due to late subacute haemorrhage. No associated retroperitoneal collection is seen.

**Figure 2:**
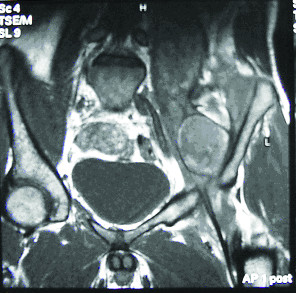
**MRI scan**. T1; Coronal image, diffuse swelling is seen involving the left iliopsoas muscle showing heterogeneous signal intensity being iso-, hypo- and hyper-intense mostly due to late subacute haemorrhage. No associated retroperitoneal collection is seen.

A 20-year-old male patient presented to our hospital in March 2005 with a six-month history of left leg pain, weakness and limping. On examination the thigh muscles were wasted, mainly the quadriceps, and the hip joint was flexed slightly with limitation of active movement in all directions. Magnetic resonance imaging (MRI) of the hip and pelvis showed a retroperitoneal mass (Figures 1 and 2), and the preliminary diagnosis was of a rhabdomyosarcoma, for which a Tru-cut biopsy was performed.

**Figure 3:**
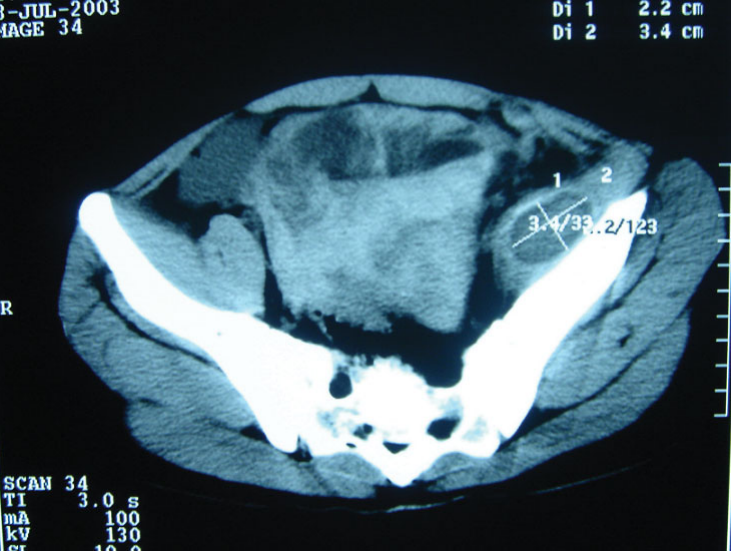
**Plain CT scan**. Diffuse swelling is seen in the left iliopsoas muscle and there is a ring-like hypodense local swelling with a high-density rim noted in the left iliacus muscle.

**Figure 4:**
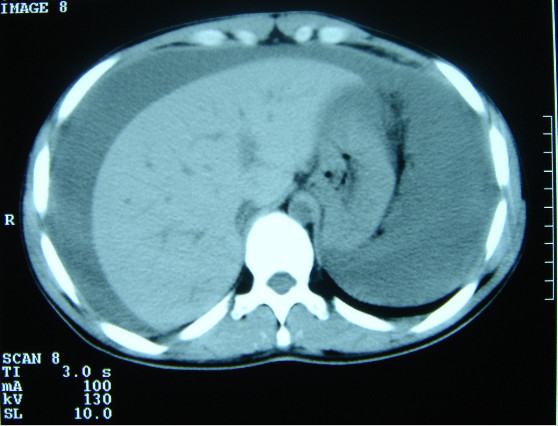
Plain CT scan of the abdomen showing intraperitoneal fluid which turned out to be haemoperitoneum.

Five days after the biopsy the patient presented to the casualty department with dizziness, abdominal pain and distension, having had one episode of haematuria. On examination, the patient was pale and his abdomen was distended, with sluggish bowel sounds. Despite the tenseness and dullness of the abdomen, tenderness was mild. Haemoglobin was 6 g/l. Urinary catheterization revealed clear urine. Ultrasound and computed tomography (CT) scan examination revealed fluid in the peritoneal cavity (Figure 3) and an iliopsoas mass (Figure 4).

The patient was resuscitated with IV fluid and blood. Given the likelihood of a vascular injury during the biopsy, an immediate exploration was planned. During laparotomy almost 2.5 l of blood was evacuated from the peritoneal cavity. A careful search failed to identify a reasonable visible source, except for a trivial amount of oozing along the Tru-cut path; this was sutured and diathermized, the mass examined and a second biopsy was taken.

The patient was reviewed the following day; haemoglobin was 12 g/l, and his other vital signs were within the normal range except for a slight temperature. Late on the postoperative day the laparotomy wound dressing was soaked with blood and needed to be changed frequently. We evacuated 300 ml of blood from the drain. This continued into the second day when further investigations were carried out with the following findings: prothrombin time (PT) 20 s (normal 14 s); partial thromboplastin time (PTT) 56.2s (normal 36.6s); bleeding, clotting time and platelets (388,000) were within the normal range. Ultrasound showed the presence of a moderate collection of intraperitoneal fluid despite the drain.

The patient received five units of fresh frozen plasma, which improved his condition slightly. Following the laboratory results the patient was asked about any past history of bruises, skin discoloration or swellings; this was the first time he had been asked about this and he confirmed these past problems. An old file was retrieved which showed that at the age of five he had been investigated for haemophilia, with no further steps taken, and neither the patient nor his parents had been updated. The diagnosis of haemophilia A was confirmed and immediately fibrinogen, cryoprecipitate and fresh frozen plasma were transfused.

The patient improved dramatically and two days later the wound was dry and the haemoperitoneum became minimal, but his PTT was still high. He was transferred to the haematology department at the university hospital, where the diagnosis of haemophilia A was confirmed, and he received Factor VIII concentrate.

## Discussion

It is well known that laboratory tests are ordered based on information obtained from the history and physical examination; in our case this was missed and, coupled with the assumed radiological diagnosis of a psoas sarcoma, all of the procedures followed this incorrect initial diagnosis [1].

There was no history taken of bruises, haemarthroses or bleeding tendency. Even after his admission to the hospital no such history was elicited; if it had been, it would have probably confirmed the presence of an underlying bleeding disorder and the diagnosis of a haemophilic pseudotumour (HP).

A bleeding disorder must be considered if the bleeding is severe or persistent, or if there is bleeding from more than one site, as in this case from the laparotomy wound. Disorders of primary haemostasis, suggesting an abnormality of platelets or small vessels, are characterized by immediate bleeding from trauma and present as petechiae or superficial ecchymoses. Conversely, impairment of secondary haemostasis (coagulation factor deficiencies) causes delayed bleeding after deep lacerations, surgery or blunt trauma, with haemorrhage into subcutaneous tissues, joints, muscles and abdominal viscera.

In the diagnosis of haemophilia, a careful history provides more valuable information than laboratory tests. Tests for specific clotting factors are not performed on all patients with a bleeding tendency, but they are certainly indicated when haemophilia is considered.

Muscular bleeding is one of the most important manifestations of haemophilia, and it is important to carry out a detailed history to eliminate any underlying bleeding disorder prior to performing any minor surgical procedure, including a biopsy. This is particularly essential in men with mild haemophilia who may remain undiagnosed until late adulthood.

There are very few cases in the literature of psoas pseudotumour. These present with weakness of a limb and the features of a lower motor neurone lesion. These pseudotumours can act as a focus for infection and, if untreated, proximal HP will ultimately destroy soft tissues, and may cause cutaneous fistulae [2,3], intraperitoneal haematomas, [4,5], erode bone or produce neurovascular complications [6]. The management of a patient with a HP is very difficult and carries a high rate of complications. There are a number of therapeutic alternatives for this dangerous condition, such as embolization, radiation or percutaneous management, but surgical excision is the treatment of choice and should only be carried out in major haemophilia centres by a multidisciplinary surgical team [2].

## Conclusion

The presence of one or more progressively enlarging masses in the pelvis of a person with haemophilia should raise the suspicion of a pseudotumour. In addition, the presence of a muscle mass in the pelvis or limb should be properly investigated and should raise the suspicion of haemophilia.

This case emphasizes three important points. The first is the need for careful evaluation of patients for an underlying coagulopathy when greater than expected bleeding occurs following a minor procedure. Second, a careful history is the most important component of the assessment for bleeding disorders in the preoperative or prebiopsy setting. The history should include questions regarding personal or family history of bleeding tendencies. A history of bleeding after dental extraction or surgery is particularly relevant. Pertinent questioning also addresses any history of haematuria, menorrhagia, gastrointestinal bleeds, easy bruising, epistaxis and hemarthroses [7]. The third point is the need for referral of these patients as soon as their condition permits to a specialized centre where facilities to deal with patients with haemophilia are available.

## Competing interests

The authors declare that they have no competing interests.

## Authors' contributions

The authors were involved in patient management or writing of the manuscript.

## Consent

Written informed consent was obtained from the patient for publication of this case report and accompanying images. A copy of the written consent is available for review by the Editor-in-Chief of this journal.
